# Case of Poisoning by Rhus Venenata

**Published:** 1891-03

**Authors:** Arthur W. Prichard

**Affiliations:** Surgeon to the Bristol Royal Infirmary


### CASE OF POISONING BY RHUS VENENATA.

BY

ARTHUR W. PRICHARD, M.R.C.S.,

*Surgeon to the Bristol Royal Infirmary.*

---

THE following case is worthy of record, on account of its rarity in this country. It is one of a serious attack of a kind of erysipelas induced by direct poison from contact with the juice of a tree called *Rhus venenata*, or "Swamp Sumach." The attack was such as I have never witnessed nor seen described in medical books. It was very severe, endangering the patient's life, and peculiar in the great disfigurement and sickening effluvium it produced. Before giving the details of the case, I will state in a few words what I have found out from botanical works and other sources about the tree.

*Rhus venenata*, belonging to the natural order *Anacardiaceæ*, is a native of North America and Canada. It is a tall-growing shrub, 15 or 20 feet high, with beautiful shining pinnate leaves, composed of 11 or 13 leaflets. The decaying leaves turn an intense red or purple, and for the beauty of its autumn tints the plant is worthy of cultivation; but care should be taken to prevent its being carelessly handled. There are 85 species of the genus Rhus, all more or less poisonous, and one of the most poisonous is the *Rhus venenata*. According to Gray's *Supplement to the Pharmacopœia*, 1847, the juice, or even air impregnated with the volatile principle of this plant, is to many persons a serious poison, producing severe and dangerous erysipelatous swellings. Khan mentions a person who by the simple exhalation was swollen to such a degree that he was as stiff as a log of wood, and could only be turned about in sheets. Some constitutions are, however, but slightly or not at all affected by it. Another authority, in the *Arboretum Britannicum*, says that the milky juice stains dark brown, and is poisonous to those who touch or smell it. In forty-eight hours inflammation appears on the skin in large blisters, principally on the extremities, attended with burning and swelling; in two or three days the eruptions suppurate. Some persons are incapable of being poisoned by it, but those who are of unstable habits are more likely to receive it. The eyes sometimes are specially affected. Japan varnish is made from the juice of this, and of one or two other species of this family; the juice being transparent at first, but becoming black on exposure to light and air.

For giving me the opportunity of seeing this case I am indebted to Mr. John Adye of Bradford-on-Avon, under whose treatment the patient made a good recovery, and who from the first recognised the cause of the attack, and who has kept careful daily notes of the whole case. The history is as follows: Mr. X., æt. 25, a curate, having his study overshadowed by a fine specimen of the tree growing in the vicarage gardens, was persuaded to cut it down. Hearing that a gardener, three years before, had suffered from blood-poisoning after cutting off a limb from the same tree, he started on the work with a certain amount of misgiving. He procured the help of the same gardener who had experienced evil effects before. After the first afternoon's work the gardener fell ill, next day suffering from an attack of erysipelas which kept him ill for a week. Mr. X. worked at the tree a short time every day for a week, at the end of which time the tree came down. He noticed that the sap, which was very profuse, made his hands black, and he subsequently wore gloves. On October 23rd some of the juice touched his face, and he wiped it off with his gloved hand. On October 24th he had three lines of redness on his cheek, which developed in two or three days' time into an attack of facial erysipelas, and this grew persistently, but not rapidly, worse. On October 30th he was so ill that he had to keep his bed: the arms, genitals, and thighs were attacked, first by a red rash, then bullæ appeared, suppurated, and burst, and large crusts formed, the exudation from under them being very profuse. His ability to take nourishment remained fairly good, and his strength kept up. The temperature never reached 103°, and his pulse did not exceed 112. The bowels were very obstinate, requiring strong purgatives before they acted; and the urine was very scanty, but not albuminous.

On the 5th of November, when I saw him, he was lying in bed with the whole of his face, throat, and neck a mass of black scabs, some of which were partly loosened by the copious purulent exudation underneath them; he was unable to see on account of the stiffness of his closed lids, and unable to masticate from the same condition of his cheeks. He could protrude the tip of his tongue, which was moist; he could swallow, and he could speak. On raising the lids, pus poured from his eyes; the conjunctivæ were swollen, but the corneæ bright and the sight good. The forearms and hands, the front and inside of the thighs, and the lower part of the abdomen were in somewhat the same condition as his face, covered with large crusts surrounded by a pink rash; but the crusts were not so black as on his face. The genitals were much swollen. The effluvium emitted by the patient was foul and peculiar. He was thirsty; but his mind was very clear. He was very querulous, complaining most of the itching of his eyes, or the burning of his skin; but he was able to sleep.

This was the day after his worst day. During the next week he slowly improved, the crusts softened and came off—those on his face last—and the conjunctivitis lessened. He was able to sit up on the 9th, and, except for the painful complication of a whitlow, he made an uninterruptedly good recovery. By the 16th he was convalescent: his eyes had ceased to discharge, he was able to read and to walk, his face and arms were peeling, and there was no pitting; the moustache had to be shaved off, as it was impossible to free it from scabs; and a fortnight later he went away for a change.

The treatment throughout the case was tonic and antiseptic. The patient, fortunately, could digest well right through his illness, and he was kept up by sustaining nutriment, stimulants, and large doses of tincture of perchloride of iron. Strong aperients had to be given when necessary; and the local applications consisted of eucalyptol, iodoform and oil, and poultices to the scabs. The eyes were treated with boracic acid lotion.

There are one or two remarks I will make about the case.

In the early part of the paper I used the expression "endangering the patient's life." I believe that to most other people such an attack would be a source of very grave danger, as at one time (Nov. 4th) such a large surface was suppurating, and the pus so profuse and, notwithstanding disinfectant applications, so fetid. He was a very robust man, and I believe he owes his life to the healthy condition of his digestive organs, which enabled him to absorb sufficient food and medicine to overcome the effects of the unwholesome surroundings in which he had to lie.

The observations of the older writers about this plant, with regard to the susceptibility of the poison by individuals, are borne out in this case by the fact that this gentleman and the gardener were the only two who were affected by the poison, although the tree was close to the house and to a tennis-lawn, and although several others joined in the work of cutting it down, or in conversation close by, and were not influenced by the poison. Mr. X. observed to me that he had always been prone to catch any illness very readily.

The specific action of the virus in the system was shown, in my opinion, by the blackness of the scabs on the parts exposed to light and air, and the varnish-like smell of the exudation, and the diminished fluid excretions, due probably to the extreme astringency of the poison.

However this may be, the rumour that the tree was so poisonous has had such an effect in the neighbourhood, that no person can be found brave enough to touch it; and there the tree lies on the vicarage lawn at Bradford, awaiting cremation when it is dry.

